# Analysis of blood coagulation process based on fractality and dynamic characteristic of laser speckle pattern

**DOI:** 10.1117/1.JBO.24.3.031018

**Published:** 2018-12-19

**Authors:** Naomichi Yokoi, Yoshihisa Aizu, Jun Uozumi

**Affiliations:** a National Institute of Technology, Asahikawa College, Department of Mechanical Systems Engineering, Asahikawa, Japan; b Muroran Institute of Technology, College of Design and Manufacturing Technology, Muroran, Japan; c Hokkai-Gakuen University, Department of Electronics and Information Engineering, Sapporo, Japan

**Keywords:** blood coagulation, fractal dimension, image processing, speckle imaging

## Abstract

The reflection and transmission of coherent light from a biological system can yield information about its condition. In the case of blood exposed to the air, there is a change in the properties of the speckle patterns observed in the coagulation process. This can be studied by means of the rate of temporal variation, the contrast, and also the fractality of patterns. The fractality of the speckle pattern can be investigated by a fractal dimension, which can quantify a level of the complexity of platelet aggregation structure and a fibrin network formed in the process of blood coagulation. In addition, dynamic characteristics of a movement in blood also contain information on the progress of the coagulation process. Fractality and dynamic characteristics are investigated simultaneously for speckle patterns observed in the coagulation process of stored horse blood. Experimental results show the feasibility of the proposed method for detecting hemolysis and formation of platelet aggregation structure and the fibrin network during the coagulation process.

## 1 Introduction

The scattering of coherent light from a biological system indicates the sign of basic physical phenomenon, such as hemodynamics and metabolism. The reflected or transmitted coherent light through a biological specimen yields a granular intensity pattern, which is called speckle. The characteristics of speckle patterns generated by blood in the coagulation process vary according to hemolysis and the formation of platelet aggregation structure and a fibrin network. Especially, a fractal dimension (FD) of the speckle patterns reflects the change in the complexity of platelet aggregation structure and a fibrin network.^1^^–^^3^ Therefore, it is expected to be an index for indicating the degree of the progress in the blood coagulation process. FD has been utilized so far for investigating atherosclerotic plaques,^4^^–^^7^ assessing the viscoelastic properties of blood during coagulation,^8^ studying the fibrin polymerization,^9^ and estimating fibrinogen levels.^10^ Recently, Yokoi et al.^11^ studied FD of speckle patterns observed in the coagulation process of blood exposed to the air.

To estimate the degree of the progress in blood coagulation, the investigation of dynamic characteristic of a movement in blood also seems to be effective. Here, the movement denotes the motion of blood cells, hematolysis, aggregation of platelets, and growth of the fibrin network. For this purpose, biospeckle fluctuations,^12^^,^^13^ which can easily be observed from biological specimens under illumination of laser light in the form of a temporal variation of laser speckle patterns, is thought to be available. The intensity fluctuation of laser light from biological specimens was first studied by Briers.^14^ Then, the visualization of retinal blood perfusion using laser speckle photography was performed by Fercher and Briers.^15^ Dunn et al.^16^ also proposed a method for dynamic imaging of cerebral blood perfusion by means of biospeckles. After that, several techniques for imaging blood perfusion based on an intensity difference in a temporal sequence of biospeckles were studied by Fujii et al.,^17^^,^^18^ Aizu et al.,^19^^–^^22^ Konishi et al.,^23^^,^^24^ and Yokoi et al.^25^^–^^34^ Especially, the average derivative (AD) proposed by Konishi and Fujii^23^ is an estimation parameter for the analysis of temporal contrast of biospeckle image analysis. Thus, AD can be used for investigating dynamic characteristic of the movement in blood.

In this study, we first investigate fractality of speckle patterns generated by the blood in the coagulation process under illumination of laser diodes (LDs) with four wavelengths by means of FD. The aim of the use of LDs with multiple wavelengths is to study the optimum wavelength with which FD can be estimated with the highest response to the progress of coagulation process in blood. For comparison purposes, FD of an incoherent transmission pattern through the blood under the illumination of a white-light source is also estimated here. We further investigate dynamic characteristics of the movement in blood by applying AD^23^ to speckle patterns and incoherent transmission patterns. Experiments are conducted for a thin layer of stored horse blood in the coagulation process to confirm the feasibility of the proposed method for estimating FD and AD simultaneously. The innovation of this study is that it investigates the coagulation process not only by spatial information but also by temporal variation information of speckle patterns. It permits to analyze the flowability of blood under the coagulation process in addition to its structure. Thus, the highlight of this study is to obtain information on spatial and temporal variation characteristics of speckle patterns generated by blood in the coagulation process at the same time. This is thought to be useful for analyzing flow characteristic of blood which is accompanied by the progressive formation of thrombin.

## 2 Principle

**Fig. 1:**
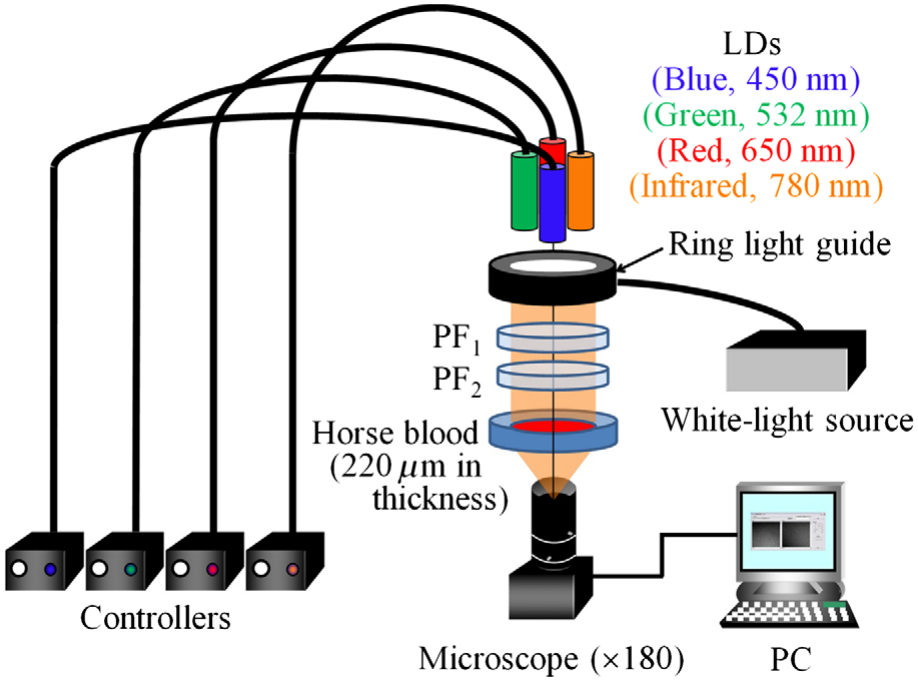
Schematic diagram of the optical system for detection of incoherent transmission patterns and speckle patterns.

Figure 1 shows a basic optical system used for detecting incoherent transmission patterns and speckle patterns. Light from the white-light source and LDs with wavelengths of 450 nm (blue), 532 nm (green), 650 nm (red), and 780 nm (infrared) passes through two polarizing filters (${\mathrm{PF}}_{1}$ and ${\mathrm{PF}}_{2}$) and then illuminates a horse blood layer of ∼220  μm in thickness. Here, the purpose of using two polarizing filters is to control the intensity of the light illuminating the blood layer. All light sources employed in this study are randomly polarized. Therefore, we first extract a linearly polarized light from a randomly polarized light using ${\mathrm{PF}}_{1}$ and, then, adjust its intensity using ${\mathrm{PF}}_{2}$. We do not intend to adjust the plane of polarization of the illuminating light by employing two polarizing filters. The transmitted light from the horse blood layer passes through a camera lens and then reaches a microscope (SKM-S30B-PC, SAITOH KOUGAKU, Japan) which has a magnification of 180. This has been confirmed by our preliminary experiments to be an optimum magnification for resolving red blood cells (RBCs), platelet aggregation structure, and the fibrin network in relatively wide field of view. A personal computer (PC) is used to analyze image data and to display FD and AD maps of the subject.

The coherence lengths of blue, green, red, and infrared LDs employed in this study are within the range of 2.0 to 6.1 mm. The correlation times of these LDs are within the range of 6.7 to 20.3 ps. The polarization state of backscattered light from the sample is nonpolarized for all LDs. The integration time of the microscope is ∼111  ms. The average speckle diameters are ∼29.7, 44.8, 57.9, and 62.9  μm for blue green, red, and infrared LDs, respectively. The coherence length and correlation time of the light source affect the size and contrast of speckles. The integration time of the microscope influences the brightness, contrast, and rate of temporal variation of speckles. The polarization state of backscattered light from the sample affects the contrast and size of speckles. The changes in absorption and scattering by the blood in the coagulation process yield the change in the brightness and contrast of speckles. Thus, these parameters interact with each other and determine temporal and spatial properties of the speckle pattern. Therefore, we have to say that it is very difficult to get robust information on fractality of speckle patterns. Nevertheless, we consider that the relative change in fractality during the coagulation process of a certain specimen of blood can be investigated by the present methods. This can be utilized, for instance, for investigating the process of formation of thrombin in blood of a patient of arteriosclerosis.

In this study, FD based on the box-counting method^35^ was used for estimating the degree of blood coagulation quantitatively. We introduce, as FD, a box dimension defined as

$$\mathrm{FD}=-\underset{r\to 0}{\lim}\frac{\log_{e}(N_{r})}{\log_{e}(r)},$$ (1)

where $N_{r}$ is the number of boxes covering a self-similar shape and r is the size of boxes. In this study, r is set to 4, 8, 16, and 32. The box-counting method counts $N_{r}$ for different values of r and plots the natural logarithm of the number $N_{r}$ versus the natural logarithm of r. The value of FD is estimated from the slope of a straight line obtained by applying the least squares method to the plotted data.

To estimate the dynamic characteristic of the movement in the blood layer, the analysis of temporal contrast of the speckle pattern is available. If there is a movement in the blood layer, the intensity of the speckle pattern fluctuates temporally and spatially. The faster the movement, the more rapidly the speckle pattern varies in intensity. When the speckle intensity pattern is sequentially recorded by the CCD camera, the signal intensity in each pixel varies from one frame to another. A rate of this variation is expected to be initially increased, reached the highest value, and then decreased with increase of the velocity of a moving object. When the rate of intensity fluctuation is roughly smaller than the frame rate of the CCD camera, AD,^23^ defined as

$$\mathrm{AD}=\frac{\frac{1}{N-1}\overset{N-1}{\underset{n=1}{\sum }}|I_{k}(t_{n+1})-I_{k}(t_{n})|}{\frac{1}{N}\overset{N}{\underset{n=1}{\sum }}I_{k}(t_{n})},$$ (2)

is effective for velocity sensing. Here, $t_{n}$ is the time corresponding to n’th frame and N is the total number of frames used for estimation. Here, N is set to 9 for all estimations of AD performed in this study. Additionally, $I_{k}(t_{n})$ is the light intensity detected at the k’th pixel of n’th frame, which is given as

$$I_{k}(t_{n})=\int_{\sigma }ds\int_{t_{n}}^{t_{n}+\Delta T}i_{k}(t,s)dt,$$ (3)

where $i_{k}(t,s)$ is the signal intensity at the k’th pixel and σ is the effective area of the detector element. Equation (2) shows that AD at pixel k is the ratio of average differences between the pair of frame data scanned successively at $t_{n}$ and $t_{n}+\Delta t$ (n=1,2,…,N) to the mean intensity. As shown in Eq. (2), AD is proportional to the average absolute difference between the pair of intensities detected at k’th pixel for two successive frames. This absolute difference value increases as the speckle pattern varies fast. AD is, thus, expected to be raised with increase of the activity of the movement in blood.

## 3 Results and Discussion

### 3.1 Analysis of the Blood Coagulation Process by Means of Fractal Dimension

**Fig. 2:**
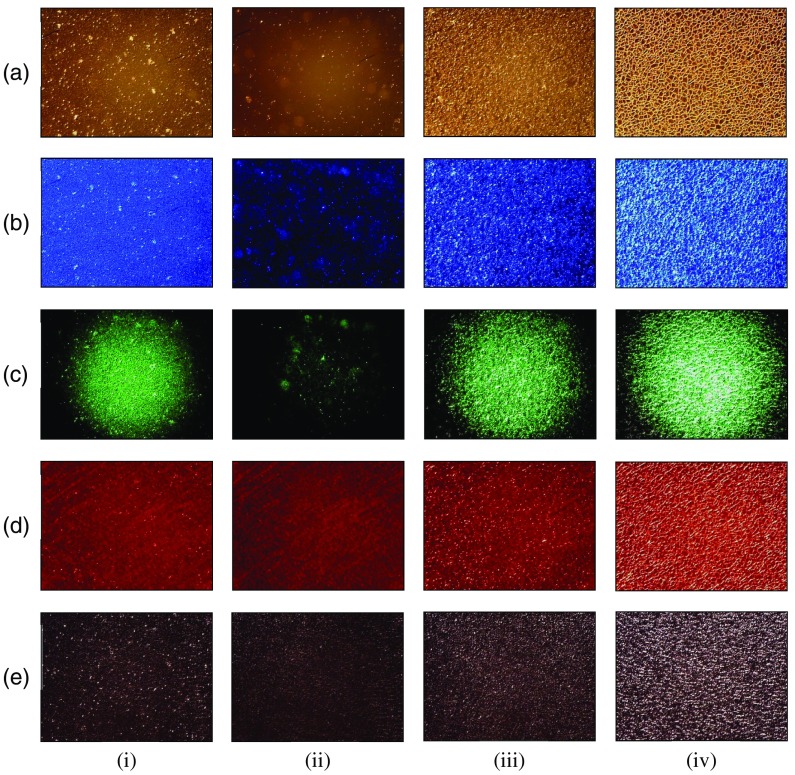
Examples of (a) incoherent transmission patterns obtained under illumination of the white-light source and (b)–(e) speckle patterns obtained under illumination of LDs with wavelengths of 450, 532, 650, and 780 nm, respectively, of the horse blood layer in coagulation process at the time of (i) 0, (ii) 31, (iii) 36, and (iv) 70 min.

We first apply FD to the analysis of the coagulation process of stored horse blood (0103-2, Nippon Bio-test Laboratories, Japan) which is employed as the subject. The total measurement time is set to 70 min. Figure 2(a) shows examples of incoherent transmission patterns of the horse blood layer in the coagulation process at the time of (i) 0 min (measurement started), (ii) 31 min (hematolysis finished), (iii) 36 min (platelet aggregation structure formed), and (iv) 70 min (fibrin network formed) obtained under illumination of the white-light source. The results in Fig. 2(a) show that a texture of the incoherent transmission pattern changes with the progress in the blood coagulation process. As shown in Fig. 2(a)(i), RBCs that look like white grains are evenly distributed over the entire sight field. It can be seen from Fig. 2(a)(ii) that both the number and size of RBCs are reduced owing to the progress of hematolysis. The result in Fig. 2(a)(iii) shows that aggregation structure of platelets is uniformly formed. Moreover, the result in Fig. 2(a)(iv) apparently displays a fine mesh structure, which is exactly the fibrin network.

Figures 2(b)–2(e) show examples of speckle patterns of the horse blood layer at same times as in Fig. 2(a) obtained under illumination of LDs with wavelengths of 450, 532, 650, and 780 nm, respectively. As shown in Figs. 2(b)–2(e), the states of hematolysis, aggregation of platelets, and formation of the fibrin network are demonstrated in the same manner as shown in Fig. 2(a), although there are some differences. At 0 min, RBCs are displayed like white grains as shown in Figs. 2(d)(i) and 2(e)(i), while they are not displayed in Figs. 2(b)(i) and 2(c)(i). This is due to that absorption of blue and green light by RBCs is larger than that of red and infrared light. Thus, RBCs are imaged like dark spots in Figs. 2(b)(i) and 2(c)(i). Additionally, speckle patterns in Figs. 2(b)(i) and 2(c)(i) also show that the average size of speckles for blue and green LDs seems to be smaller than that for other light sources. This is due to that platelets inside the blood layer are well resolved by smaller speckles originate from narrower point spread function (PSF) of the speckle intensity distribution owing to the smaller wavelength of illuminating light. At 31 min, the brightness of speckle patterns in Figs. 2(b)(ii) and 2(c)(ii) seems to be lower than that of the incoherent transmission pattern in Fig. 2(a)(ii). This is caused by the significant decrease in speckle intensity owing to larger absorption of blue and green light by hemoglobin eluted from RBCs in comparison with other light. At 36 min, speckle patterns in Figs. 2(b)(iii)–2(e)(iii) evenly display aggregates of platelets in the same manner as the incoherent transmission pattern in Fig. 2(a)(iii) does. Especially, at 70 min, speckle patterns in Figs. 2(b)(iv)–2(e)(iv) distinctly display the structure of the fibrin network in the same manner as the incoherent transmission pattern in Fig. 2(a)(iv) does.

**Fig. 3:**
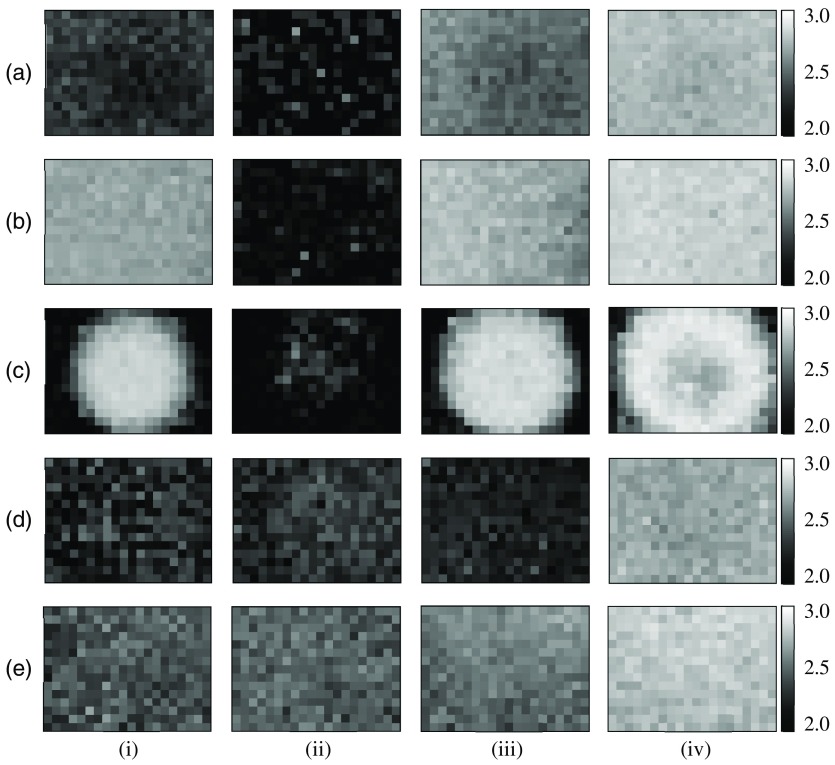
Examples of FD images of the horse blood layer in coagulation process obtained under illumination of (a) white-light source and (b)–(e) LDs with wavelengths of 450, 532, 650, and 780 nm, respectively, at the time of (i) 0, (ii) 31, (iii) 36, and (iv) 70 min.

Figures 3(a)–3(e) show examples of 8-bit grayscale images of FD of the horse blood layer in the coagulation process obtained for the white-light source and LDs with wavelengths of 450, 532, 650, and 780 nm, respectively, at the time of (i) 0, (ii) 31, (iii) 36, and (iv) 70 min. Here, each image in Fig. 3 was calculated from the incoherent transmission pattern or the speckle pattern as shown in Fig. 2. As shown in Fig. 3, the brightness of the FD image seems to be decreased at first and then increased with the progress of the blood coagulation process for all illuminations. At 0 min, the brightness of FD images in Figs. 3(b)(i) and 3(c)(i) seems to be higher than that of other images. This is caused by the increase in complexity of speckle patterns owing to the enhanced resolution of platelets using illumination of LDs with shorter wavelengths. At 31 min, the brightness of FD images in Figs. 3(d)(ii) and 3(e)(ii) seems to be higher than that of other images. This is due to that the absorption of red and infrared light by hemoglobin eluted from RBCs is smaller than that of other light sources and, thus, speckles that originate from platelets are fully observed. At 36 min, the brightness of FD image in Fig. 3(d)(iii) is apparently lower than that of other images. This may be caused by the low resolution of the platelet aggregation structure under illumination of red LD owing to properties of scattering and absorption of red light by aggregate of platelets. At 70 min, the brightness of FD images in Figs. 3(a)(iv)–3(e)(iv) seems to be evenly high owing to the formation of the fibrin network shown in Figs. 2(a)(iv)–2(e)(iv). The fractality of speckle patterns in Figs. 2(b)(iv)–2(e)(iv) derives from the fractal network of the aggregated fibrin as shown in Fig. 2(a)(iv) and is due to that speckle patterns resolve the fractal structure to a certain degree.

**Fig. 4:**
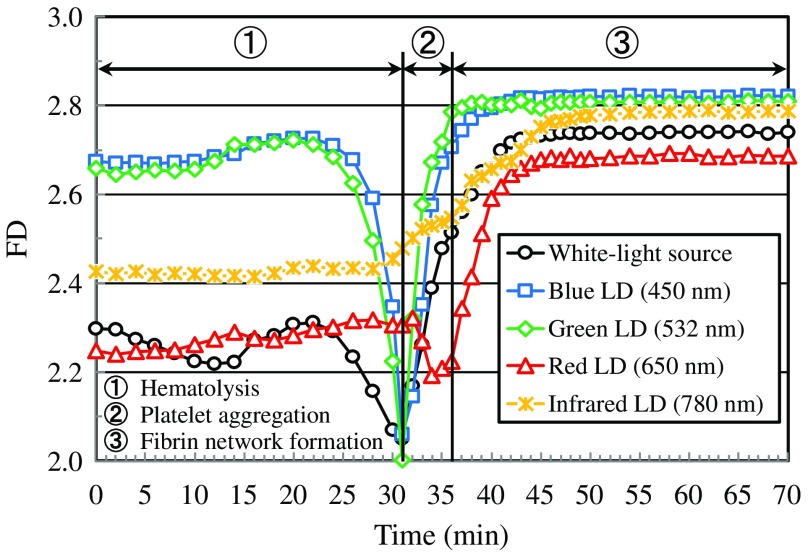
AD value versus elapsed time relations obtained for the white-light source and LDs with wavelengths of 450, 532, 650, and 780 nm.

Figure 4 shows FD value averaged over each FD image obtained under illumination of the white-light source and LDs as shown in Fig. 3 as a function of elapsed time. Here, the area used for averaging FD was the entire image except for the case of the green LD. For the green LD, brightness data in the marginal and central areas of the image were excluded in averaging FD for avoiding the influence of unevenness of the image on averaged FD value. This unevenness of the FD image is caused by the nonuniformity of the speckle intensity as shown in Fig. 2(c), which is due to the intensity irregularity of the illuminating light. As for the plotted data in Fig. 4, the average standard deviation values of FD are 0.0008, 0.0011, 0.0013, 0.0021, and 0.0015 for white-light source and blue, green, red, and infrared LDs, respectively. The results in Fig. 4 show that FD in the early and middle stages of hematolysis takes high value for blue and green LDs owing to the high resolution of platelets. At the same time, FD takes high value even for the infrared LD. This result is in contradiction with the expectation that wider PSF gives larger speckles and gives lower resolution of RBCs and platelets. Here, the scattering and absorption of light by RBCs should also be taken into consideration. The scattering and absorption of the infrared light by hemoglobin in RBCs are smaller than those of blue, green, and red light.^36^ Therefore, light entering the blood layer is highly transmitted, and speckles originating from RBCs and platelets are imaged with high signal-to-noise ratio. Thus, FD for infrared light takes comparatively high value owing to higher signal-to-noise ratio of speckles originating from RBCs and platelets in the blood layer. The results in Fig. 4 also show that FD significantly decreases in the end stage of hematolysis for blue and green LDs and the white-light source. This is due to vanishing RBCs, which makes images of speckle pattern and incoherent transmission pattern smoothed and reduces their complexity. Additionally, the significant decrease in the speckle intensity owing to large absorption of blue and green light by hemoglobin eluted from RBCs also contributes to the decrease in FD. This decrease is, however, not observed under illumination of red and infrared LDs. This is due to the smaller absorption of red and infrared light by hemoglobin eluted from RBCs in comparison with other light sources. It can also be seen from results in Fig. 4 that FD significantly increases during platelet aggregation for illumination of light sources except for the red LD. This fact suggests that the complexity of the speckle pattern is increased in accordance with the aggregation of platelets. The reduction of FD during platelet aggregation under illumination of red LD may be caused by properties of scattering and absorption of red light by aggregate of platelets. Moreover, results in Fig. 4 show that FD continuously increases in an early stage of fibrin network formation and then reaches a plateau under illumination of all light sources. This reproduces the process of progress and completion of formation of the fibrin network. Furthermore, it is remarkable in the results in Fig. 4 that FD values for blue and green LDs are higher than that for the white-light source for the whole coagulation process. For blue and green LDs, speckles superimposed on the fractal network pattern of the aggregated fibrin may raise the FD value. It should also be noticed here that the rate of progress of blood coagulation is dependent on conditions such as a kind of blood, thickness of the blood layer, blood concentration, temperature, and humidity. We confirmed by our preliminary experiments that the progress of the coagulation process was affected by the surrounding environments, such as temperature environment and humidity environment, even if conditions of blood were set to be identical.

### 3.2 Analysis of Dynamic Characteristic of the Movement in Blood by Means of Average Derivative

**Fig. 5:**
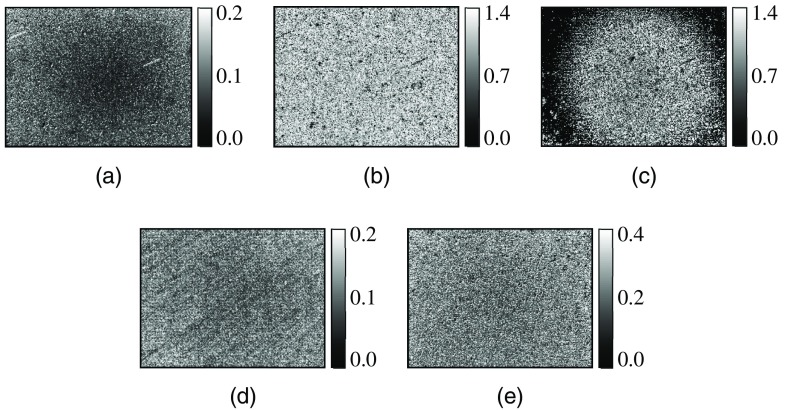
Examples of FD images obtained for (a) white-light source and (b)–(e) LDs with wavelengths of 450, 532, 650, and 780 nm, respectively, at 14 min.

We next apply AD to the analysis of dynamic characteristic of the movement in the horse blood layer in the coagulation process. Figures 5(a)–5(e) show examples of 8-bit grayscale images of AD of the horse blood layer at 14 min (during hematolysis) for illumination of the white-light source and blue, green, red, and infrared LDs, respectively. Here, each image in Fig. 5 was calculated from the incoherent transmission pattern or the speckle pattern as shown in Fig. 2. As seen from results in Fig. 5, AD images obtained for blue and green LDs are brighter than those obtained for other light sources. Although light from blue and green LDs are efficiently absorbed by RBCs in the blood layer, the density of RBCs is not as high as seen in the incoherent transmission pattern in Fig. 2(a)(i). Additionally, a smaller wavelength of the illuminating light source gives higher scattering of water which occupies the greater part of the blood layer during hematolysis. Therefore, the light from both the LDs can pass through a space between RBCs while being highly scattered by water and platelets in the blood layer. Then, they generate speckles that can reflect the movement in the blood layer more effectively than other light sources. Thus, AD values obtained for blue and green LDs become higher than those obtained for other light sources.

**Fig. 6:**
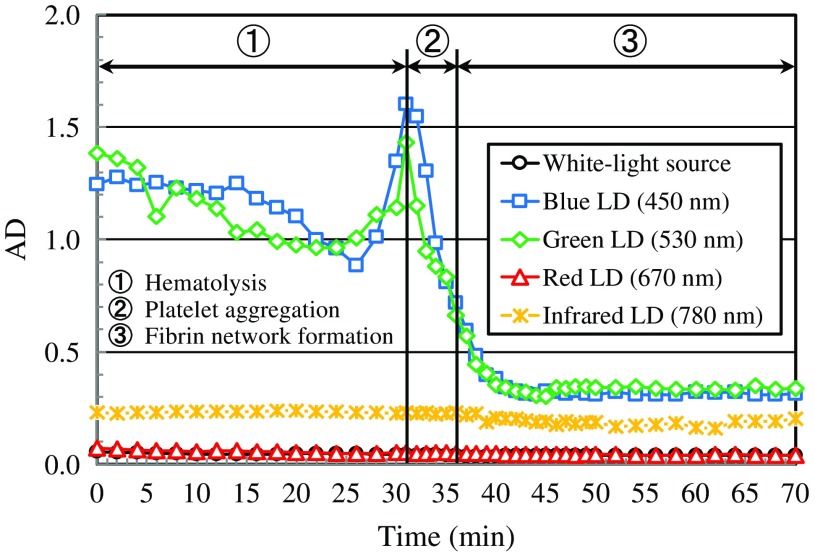
FD value versus elapsed time relations obtained for the white-light source and LDs with wavelengths of 450, 532, 650, and 780 nm.

Figure 6 shows AD value averaged over each AD image obtained under illumination of the white-light source and LDs with four wavelengths as shown in Fig. 5 as a function of elapsed time. As seen from results in Fig. 6, AD values obtained for blue and green LDs are higher than those obtained for other light sources over the whole measurement time. This fact implies that blue and green LDs can effectively reproduce the change in activity of the movement in blood during the coagulation process. The results in Fig. 6 also show that AD values for blue and green LDs gradually decrease from 0 to 24 min, then drastically increase from 26 to 31 min and, suddenly decrease after 32 min. The gradual decrease of AD value from 0 to 24 min is thought to be caused by the increase in blood viscosity owing to the effect of hemoglobin eluted from RBCs by hematolysis. The drastic increase of AD value in the end stage of hematolysis may be due to the platelet activation, which generally occurs in advance to aggregation of platelets. The sudden decrease of AD after 32 min is thought to be caused by the fact that platelets and fibrins in the blood layer are immobilized owing to the formation of platelet aggregation structure and the fibrin network. Thus, AD values for blue and green LDs have the ability to reflect the process of blood coagulation faithfully.

## 4 Conclusion

In summary, we have investigated fractality in combination with the dynamic characteristic of the speckle pattern observed in the coagulation process of blood. We first estimated FD of the speckle pattern generated by the horse blood layer under illumination of LDs with four wavelengths and clarified that fractality of the speckle pattern is increased in accordance with aggregation of platelets and growth of the fibrin network. This fact suggests that fractality of speckle patterns reproduces the blood coagulation process in the same manner as that of incoherent transmission patterns does from the past. A disadvantage of FD is that it reduces the spatial resolution of the resultant blood coagulation map in comparison with the original pattern. A possible solution to this problem is the use of a high-resolution CCD camera. We next investigated dynamic characteristic of the movement in the horse blood layer under the coagulation process by means of AD and confirmed that blue and green LDs reflected the process of blood coagulation faithfully. The most serious problem in the estimation of AD was the influence of noises included in the speckle pattern. This problem may be solved using a high-sensitive image sensor.

Altogether, fractality and the dynamic characteristic of speckle patterns generated by the blood layer can simultaneously be estimated by employing blue and green LDs as light sources in case of the transmission type measurement. This permits us to obtain information on spatial and temporal variation characteristics of speckle patterns generated by blood in the coagulation process at the same time. This may be, for instance, utilized for analyzing the flow characteristic of blood which is accompanied by the progressive formation of thrombin. For future development, the present method may be applied to prevention of arteriosclerosis, elucidation of generation mechanism of economy class syndrome (occurrence of deep vein thrombosis in air travelers), and monitoring of the progress state of hemostasis, for instance.
